# Gestational and Chronic Low-Dose PFOA Exposures and Mammary Gland Growth and Differentiation in Three Generations of CD-1 Mice

**DOI:** 10.1289/ehp.1002741

**Published:** 2011-04-18

**Authors:** Sally S. White, Jason P. Stanko, Kayoko Kato, Antonia M. Calafat, Erin P. Hines, Suzanne E. Fenton

**Affiliations:** 1 National Toxicology Program, National Institute of Environmental Health Sciences, National Institutes of Health, Department of Health and Human Services, Research Triangle Park, North Carolina, USA; 2 Division of Laboratory Science, National Center for Environmental Health, Centers for Disease Control and Prevention, Atlanta, Georgia, USA; 3 National Center for Environmental Assessment, Office of Research and Development, U.S. Environmental Protection Agency, Research Triangle Park, North Carolina, USA

**Keywords:** delayed development, fetal origins of adult disease, lactation, mammary gland, multigenerational, perfluorooctanoic acid (PFOA)

## Abstract

Background: Prenatal exposure to perfluorooctanoic acid (PFOA), a ubiquitous industrial surfactant, has been reported to delay mammary gland development in female mouse offspring (F_1_) and the treated lactating dam (P_0_) after gestational treatments at 3 and 5 mg PFOA/kg/day.

Objective: We investigated the consequences of gestational and chronic PFOA exposure on F_1_ lactational function and subsequent development of F_2_ offspring.

Methods: We treated P_0_ dams with 0, 1, or 5 mg PFOA/kg/day on gestation days 1–17. In addition, a second group of P_0_ dams treated with 0 or 1 mg/kg/day during gestation and their F_1_ and F_2_ offspring received continuous PFOA exposure (5 ppb) in drinking water. Resulting adult F_1_ females were bred to generate F_2_ offspring, whose development was monitored over postnatal days (PNDs) 1–63. F_1_ gland function was assessed on PND10 by timed-lactation experiments. Mammary tissue was isolated from P_0_, F_1_, and F_2_ females throughout the study and histologically assessed for age-appropriate development.

Results: PFOA-exposed F_1_ dams exhibited diminished lactational morphology, although F_1_ maternal behavior and F_2_ offspring body weights were not significantly affected by P_0_ treatment. In addition to reduced gland development in F_1_ females under all exposures, F_2_ females with chronic low-dose drinking-water exposures exhibited visibly slowed mammary gland differentiation from weaning onward. F_2_ females derived from 5 mg/kg PFOA-treated P_0_ dams displayed gland morphology similar to F_2_ chronic water exposure groups on PNDs 22–63.

Conclusions: Gestational PFOA exposure induced delays in mammary gland development and/or lactational differentiation across three generations. Chronic, low-dose PFOA exposure in drinking water was also sufficient to alter mammary morphological development in mice, at concentrations approximating those found in contaminated human water supplies.

Perfluorooctanoic acid (PFOA) is a fully fluorinated eight-carbon perfluoroalkyl acid (PFAA) with a carboxylic acid functional group. As with other PFAAs, PFOA is used in the production of fluorochemicals, which have extensive commercial applications (Prevedouros et al. 2006). PFOA is also a final breakdown product of certain fluorochemicals and resists degradation in the ambient environment by biota or physical processes (Martin et al. 2005). The ubiquity of fluorochemicals in the marketplace, combined with the persistence of PFOA in the environment, may explain current widespread PFOA contamination of humans and wildlife (Giesy and Kannan 2002; Harada et al. 2004; Martin et al. 2004).

The average nonoccupationally exposed American exhibits measurable serum PFOA, varying between a mean concentration of 3.9 ng/mL among participants in the 2003–2004 National Health and Nutrition Examination Survey (Calafat et al. 2007) and 2.2 ng/mL in 2005 among a smaller group of Red Cross blood donors (Olsen et al. 2007). Occupational exposure can raise serum concentrations more than 200 times this approximate range (Emmett et al. 2006). In the Little Hocking district of Ohio and West Virginia where the municipal drinking-water supply was contaminated with PFOA at 3.55 ng/mL (ppb) by nearby production plants, mean human serum concentrations were 423 ng/mL (Emmett et al. 2006). Thus, nonoccupationally exposed Americans may receive substantial unforeseen exposures to PFOA. It is not known, however, whether adverse adult health effects could result from these chronic, low-level exposures beginning in early life. This is of particular interest with respect to development, because the potential toxicity of PFOA in humans remains uncharacterized.

Mouse studies have demonstrated the capacity for gestational PFOA exposure to yield developmental toxicity (Lau et al. 2004, 2006; Wolf et al. 2007). The mammary gland, specifically, has proven to be a sensitive tissue with respect to the developmental end points addressed, including functional lactation, milk protein gene expression, and developing neonatal and peripubertal structures (White et al. 2007, 2009; Yang et al. 2009; Zhao et al. 2010). In outbred CD-1 mice, treatment with 3 mg/kg PFOA during pregnancy resulted in delayed gland development among offspring, which persisted into adulthood, even among offspring with lactational exposures only (White et al. 2009). Another laboratory examined similar dose ranges using peripubertal exposures [postnatal days (PNDs) 21–50] in two inbred mouse strains, C57Bl/6 and Balb/C. The researchers observed a similar inhibitory effect on mammary gland development in Balb/C mice (Yang et al. 2009), whereas C57Bl/6 females exhibited stimulatory or inhibitory effects depending on dose (Yang et al. 2009; Zhao et al. 2010). These observations illustrate the influence not only of dose but also of exposure timing and genetic background. They confirm that the mammary gland represents a sensitive tissue in multiple mouse strains.

To understand the extended consequences of altered mammary gland development, we performed a multigenerational study examining the ability of the developmentally exposed females to provide lactational support for their litters. To address the human relevance of the route, dose, and duration of exposures employed in our studies, we included a chronic low-dose exposure.

## Materials and Methods

*Animals.* Timed-pregnant CD-1 mice were purchased from Charles River Laboratories (Raleigh, NC). Sperm-positive females [gestational day (GD) 0] were weighed upon arrival at the U.S. Environmental Protecion Agency (EPA). Animals were housed individually in polypropylene cages, and received food (LabDiet 5001; PMI Nutrition International LLC, Brentwood, MO) and tap water *ad libitum* in polyethylene water bottles sealed with rubber stoppers and stainless-steel sipper tubes, as specified by White et al. (2009). Animal protocols were approved by the U.S. EPA’s Institutional Animal Care and Use Committee. Animals were treated humanely and with regard for alleviation of suffering.

*Dosing solutions.* PFOA (ammonium perfluorooctanoate; > 98% pure) was purchased from Fluka Chemical (Steinheim, Switzerland). PFOA was dissolved by agitation in deionized water at concentrations of 0.1 and 0.5 mg/mL (for 1 and 5 mg/kg doses, respectively) and prepared fresh daily, immediately before administration. PFOA-containing drinking water was prepared similarly, by serial dilution to a final concentration of 5 ng/mL (ppb). Drinking water was prepared fresh weekly, and cage bottles were refilled weekly after rinsing.

*Study design.* A study timeline is shown in Supplemental Material, Figure 1 (doi:10.1289/ehp.1002741). Timed pregnant P_0_ (parental generation) dams were randomly distributed among five treatment groups. Three groups were treated once daily by oral gavage on GDs 1–17 (designated “gestational”) with PFOA doses of 0 (control; *n* = 10), 1 (*n* = 12), or 5 mg/kg body weight (*n* = 11). The remaining two groups received PFOA at 0 (*n* = 7) or 1 mg/kg (*n* = 10) as described above, but also received PFOA (5 ppb) in their drinking water (designated “chronic”) to approximate the 3.55 ppb PFOA present in the contaminated drinking- water supply in Little Hocking, Ohio (Emmett et al. 2006). These two groups received PFOA-containing drinking water throughout gestation (starting on GD7) and for the duration of the study, as did subsequent F_1_ and F_2_ offspring (except during F_1_ breeding and early gestation, to avoid exposing control males). Weekly water consumption was calculated per cage by weighing bottles when filled and again at the end of the week; the differential reflected consumption.

**Figure 1:**
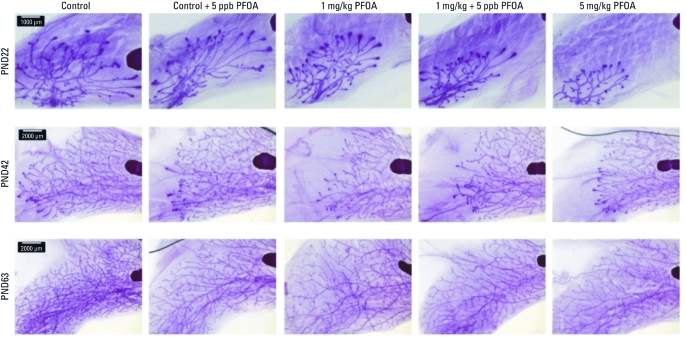
F_1_ female mammary gland development. Mammary whole-mounts illustrate morphology representative of treatment groups at PNDs 22, 42, and 63 (*n* = 6–7 females/treatment/age). Bars = 1,000 μm for PND22 and 2,000 μm for PND42 and PND63.

P_0_ dams were weighed daily throughout gestation. On PND1, F_1_ litters were weighed and sexed. F_1_ neonates were pooled and randomly redistributed to dams of their respective treatment groups, consistent with previous studies (Lau et al. 2006; White et al. 2009), equalizing litters to 12–13 neonates, with similar sex representation. Litters were monitored and weighed on PND10. On PND22, F_1_ offspring were weaned, and dams and 1–2 female offspring/litter were weighed and necropsied (*n* = 5–7 litters/treatment  group). A subset of F_1_ females were maintained into adulthood and weighed and necropsied at PND42 and PND63 (*n* = 6–8/treatment group).

Remaining adult F_1_ females were bred to age-matched control F_1_ males at 7–8 weeks of age, on the night of proestrus (determined by vaginal cytology). Breeding pairs remained together overnight only, and plug-positive females (GD0) were housed individually and monitored over gestation (*n* = 7–10 F_1_ dams/treatment group). On PND1, F_2_ neonates were weighed and sexed. F_2_ litters were equalized to 10 neonates for the lactational challenge experiment. F_1_ dams and 3 female offspring per F_2_ litter were sacrificed on either PND10 or PND22. The remaining F_2_ females were weaned and necropsied on either PND42 or PND63 (*n* = 4–8/treatment group).

The lactational challenge experiment was performed with F_1_ dams and their F_2_ litters on PND10, the peak of lactation. Dams were separated from offspring for 3 hr and then returned to their litters and allowed to nurse for 30 min. The time between reunion and initiation to nurse (arched back position over the litter) was recorded to the nearest second, as was the weight of the 10-pup litter before and after precisely 30 min of nursing, in order to estimate the volume of milk produced during the nursing period. Dams were euthanized and necropsied immediately after nursing.

*Necropsy.* All animals were terminated by decapitation; trunk blood was collected and serum was isolated and stored at –80°C in snap-top polypropylene tubes for PFOA analysis. Uteri were dissected from P_0_ and F_1_ dams, and implantation sites were visually identified by light macroscope (Leica WILD M420 macroscope; Leica, Wetzlar, Germany) to assess postimplantation loss per dam. Mammary glands were collected as described below.

*Mammary gland preparation.* Mammary glands were removed from P_0_ and F_1_ dams on PND10 (F_1_ dams only) and PND22 (*n* = 4–12/treatment group) because these times represent peak lactational output and weaning, respectively. In F_1_ and F_2_ offspring, a set of fourth and ﬁfth glands was removed from the skin and ﬂattened onto glass slides. Whole-mounts were ﬁxed in Carnoy’s solution, stained in carmine alum, and then dehydrated and cleared in xylene, as previously described (Fenton et al. 2002). From dams only, a portion of the contralateral mammary gland was removed, placed in a histology cassette, ﬁxed in 10% neutral buffered formalin for 48 hr, and stored in 70% ethanol. These tissues were embedded in parafﬁn, and 5 μm sections were prepared and stained with hematoxylin and eosin (H&E). Whole-mounts and histological sections were visualized by light macroscope.

Mammary gland whole-mounts from F_1_ and F_2_ female offspring were scored on a 1–4 subjective, age-appropriate developmental scale (4 = excellent development/structure; 1 = poor development/structure). The number of primary ducts and large secondary ducts, lateral side branching, appearance of budding from the ductal tree, and longitudinal outgrowth of the epithelia were assessed. Because we did not address estrous cycle stage at the time of necropsy, we did not include stage-sensitive morphological traits in scoring criteria. Slides were separated by score during evaluation, compared within a score for consistency, and then recorded. Two individuals, blind to treatment, scored glands. Mean scores for the various ages and treatment groups were calculated and analyzed statistically for treatment and time-related differences.

H&E-stained lactating mammary gland sections from P_0_ and F_1_ dams were similarly scored on a 1–4 subjective scale. A value of 4 represented well-differentiated, functionally lactating tissue characterized by extensive epithelium, reduced adiposity, and presence of secretory alveoli, consistent with the peak of lactation (PND10, as previously described by Vorderstrasse et al. 2004). A value of 1 represented little or diminishing presence of lobuloalveoli and extensive involution and regression of the tissue, with the presence of apoptotic bodies, increasing adiposity, and regressing alveoli, as anticipated at weaning (PND22). At both time points, dams were euthanized immediately after removal from litters to ensure comparable lactational morphology. Mammary glands representing the mean score or observation for each treatment group were photographed using the described macroscope and mounted camera (Photometrics CoolSNAP; Roper Scientiﬁc, Inc., Tucson, AZ).

*Measurement of PFOA in serum.* Serum samples from the P_0_ and F_1_ dams at PND22 and from F_1_ and F_2_ offspring at PNDs 22, 42, and 63 were stored frozen in snap-top polypropylene vials until they were shipped on dry ice to the Centers for Disease Control and Prevention (CDC) laboratory. Serum PFOA measurements were performed by the CDC using the methodology described in detail by White et al. (2009).

*Data analysis.* Data were evaluated for dose effects using mixed-model analysis of variance in SAS (version 9.1; SAS Institute Inc., Cary, NC). For both generations, treatment-specific mean gestational weight gain was calculated for dams between GD1 and GD17, and treatment-specific mean body weights were determined for F_1_ and F_2_ offspring on PNDs 22, 42, and 63. In addition, we calculated F_2_ offspring body weight means at PNDs 1, 3, 5, 10, 14, and 17, based on whole-litter weights (divided by number of pups; litter used as the unit of measure before weaning). For all three generations, mean mammary gland lactational or developmental scores were calculated. Scores were analyzed using body weight at time of collection as a random effect, with litter as the unit of measure for neonatal scores. For both P_0_ and F_1_ dams, we calculated mean implant number, percentage of postimplantation (prenatal) loss, and percentage of postnatal survival. Differences between treatment groups were determined using Dunnett’s, Tukey’s, or Student’s *t*-tests, with significance set at *p* < 0.05 for all comparisons.

## Results

*P_0_ dams and F_1_ offspring.* We found no significant effect of PFOA on P_0_ dam gestational weight gain or implant number (Table 1). Consistent with previous studies (White et al. 2007, 2009; Wolf et al. 2007), gestational 5 mg/kg PFOA significantly reduced the number of live fetuses, prenatal survival, and postnatal offspring growth and survival, but similar effects were not observed with 1 mg/kg PFOA or with drinking-water treatment (Table 1). Given these observations in P_0_ dams—and in agreement with the conclusions of prior studies (Lau et al. 2006; Wolf et al. 2007)—maternal toxicity was not responsible for F_1_ developmental deficits seen at low exposures.

**Table 1:** P_0_ maternal indices (mean ± SE; *n* = 7–11).

Maternal index	Control	Control + 5 ppb PFOA	1 mg/kg	1 mg/kg + 5 ppb PFOA	5 mg/kg
Gestational weight gain (g)		24.8 ± 1.2		25.0 ± 1.2		26.0 ± 1.2		27.0 ± 1.2		27.7 ± 1.2
Implants (no. per live litter)		12.8 ± 0.5		12.7 ± 0.4		13.5 ± 0.7		14.0 ± 0.4		13.7 ± 0.6
Live fetuses (no. per live litter)		12.0 ± 0.5		11.7 ± 0.4		12.9 ± 0.7		13.3 ± 0.5		10.0 ± 0.8*
Prenatal loss (% per live litter)		6.1 ± 1.8		7.8 ± 1.7		4.5 ± 1.7		5.1 ± 1.6		25.8 ± 5.6*
Postnatal survival (% per live litter)		96.1 ± 1.3		100 ± 0.0*		98.8 ± 0.8		89.5 ± 6.4		72.7 ± 5.8*
Mammary gland score (1–4 scale), PND22		2.4 ± 0.2		3.4 ± 0.1*		3.0 ± 0.2*		3.2 ± 0.2*		3.9 ± 0.1*
*p < 0.05 compared with control.										

As evidenced by significantly elevated histological scores at PND22, normal weaning-induced mammary involution was compromised among all PFOA-treated P_0_ dams, including those with only low-dose exposures via drinking water (Table 1). In contrast with the extensive gland regression observed in control dams at weaning, glands in PFOA-treated dams at PND22 demonstrated structural similarity to normal dam mammary tissue at or near the peak of lactation at PND10, including the presence of functional lobuloalveolar units (data not shown). This observation was consistent with our previous finding that gestational PFOA exposure delays lactational differentiation and eventual involution in the exposed dam (White et al. 2007), but here we also observed the effect with exposure to 5 ppb PFOA in drinking water for a total of 34 days [for dose estimates, see Supplemental Material, Table 1 (doi:10.1289/ehp.1002741)].

F_1_ offspring body weights and adjusted body weights (body weight less liver weight) between PND22 and PND63 were not consistently associated with PFOA treatment (Table 2). Liver:body weight ratios at PND22 were significantly elevated among F_1_ females exposed to 1 or 5 mg/kg, consistent with hepatomegaly. At PND42, F_1_ females exposed to 5 mg/kg had significantly increased liver:body weight ratios and significant reductions in total and adjusted body weight, but all three parameters were similar to controls by 9 weeks of age (PND63). Chronic 5 ppb PFOA exposure in drinking water did not affect the liver:body weight ratio in F_1_ offspring. In contrast, developmental mammary scores of F_1_ offspring were significantly reduced among all treatment groups (including 5 ppb in water) until at least 9 weeks of age (PND63; Table 2, Figure 1), suggesting that delayed mammary gland development is a more sensitive and persistent end point than is hepatomegaly.

**Table 2:** F_1_ developmental indices (mean ± SE; *n* = 4–10).

Developmental index	Control	Control + 5 ppb PFOA	1 mg/kg	1 mg/kg + 5 ppb PFOA	5 mg/kg
Body weight (g)										
PND22		12.70 ± 0.69		12.69 ± 0.87		13.40 ± 0.49		13.20 ± 0.37		11.28 ± 0.45
PND42		25.65 ± 0.43		24.28 ± 0.57		24.24 ± 0.74		24.90 ± 0.62		22.28 ± 0.60*
PND63		28.77 ± 0.96		26.23 ± 1.81		29.93 ± 0.97		26.35 ± 0.84^#^		27.88 ± 1.25
Liver:body weight ratio (×100%)										
PND22		5.56 ± 0.16		5.29 ± 0.13		6.35 ± 0.08*		5.96 ± 0.12		7.81 ± 0.34*
PND42		5.19 ± 0.24		5.75 ± 0.22		5.32 ± 0.10		5.26 ± 0.13		5.79 ± 0.09*
PND63		4.85 ± 0.17		4.99 ± 0.12		4.97 ± 0.13		4.82 ± 0.15		5.24 ± 0.28
Body weight excluding liver weight (g)										
PND22		11.99 ± 0.64		11.16 ± 0.86		12.55 ± 0.46		12.55 ± 0.36		10.39 ± 0.39
PND42		24.32 ± 0.44		22.89 ± 0.54		22.94 ± 0.69		23.59 ± 0.58		20.99 ± 0.57*
PND63		27.38 ± 0.94		24.92 ± 1.74		28.49 ± 1.12		24.43 ± 1.09		26.43 ± 1.24
Mammary gland score (1–4 scale)										
PND22		3.8 ± 0.1		2.5 ± 0.2*		2.3 ± 0.2*		2.2 ± 0.1*		1.6 ± 0.1*
PND42		3.8 ± 0.1		3.3 ± 0.2*		2.6 ± 0.4*		2.2 ± 0.3*		2.3 ± 0.2*
PND63		3.8 ± 0.2		2.6 ± 0.4*		2.9 ± 0.2*		2.0 ± 0.3*^#^		2.2 ± 0.2*
*p < 0.05 compared with control. ^#^*p* < 0.05 compared with 1 mg/kg.										

*F_1_ dams and F_2_ offspring.* We did not observe maternal toxicity in F_1_ dams with developmental or chronic low-level PFOA exposures. Interestingly, the number of uterine implants was significantly reduced among F_1_ dams developmentally exposed to 5 mg/kg, resulting in litters with significantly fewer offspring (Table 3). As previously described, postnatal survival of 5 mg/kg F_1_ females was significantly decreased; however, we observed no effect on this end point with respect to postnatal survival of F_2_ offspring. This suggests that both F_2_ thriftiness—specifically referring to the ability to suckle with sufficient vigor and frequency, so as to yield nourishment—and F_1_ lactational competency were sufficient to support litters.

**Table 3:** F_1_ maternal indices (mean ± SE; *n* = 4–10).

Maternal index	Control	Control + 5 ppb PFOA	1 mg/kg	1 mg/kg + 5 ppb PFOA	5 mg/kg
Implants (no. per live litter)		14.9 ± 0.4		14.6 ± 0.5		14.1 ± 0.4		13.4 ± 0.9		12.3 ± 0.2*
Live fetuses (no. per live litter)		13.6 ± 0.6		13.1 ± 0.6		12.8 ± 0.6		12.1 ± 0.9		12.0 ± 0.3*
Prenatal loss (% per live litter)		8.6 ± 2.5		9.8 ± 3.2		10.0 ± 3.2		6.7 ± 2.5		2.7 ± 1.4
Postnatal survival (% per live litter)		100 ± 0.0		100 ± 0.0		98.1 ± 1.4		97.9 ± 1.5		100 ± 0.0
Lactational challenge										
Milk produced in 30 min (g)		2.10 ± 0.20		1.80 ± 0.35		2.08 ± 0.25		1.40 ± 0.44		1.73 ± 0.51
Time to initiate (sec)		267 ± 38		384 ± 55		307 ± 114		351 ± 86		279 ± 30
Mammary gland score (1–4 scale)										
PND10		4.0 ± 0.0		2.8 ± 0.5*		2.5 ± 0.2*		2.0 ± 0.2*		2.5 ± 0.2*
PND22		2.1 ± 0.3		2.2 ± 0.2		1.9 ± 0.4		1.5 ± 0.2*		3.2 ± 0.3*
*p < 0.05 compared with control.										

**Figure 2:**
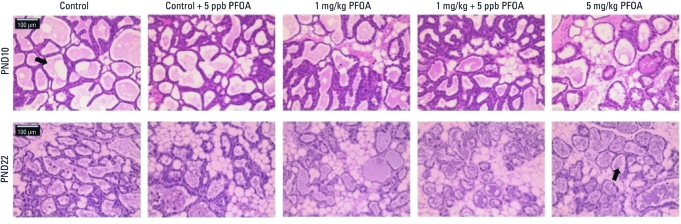
Histological sections of lactating mammary glands from F_1_ dams. Glands pictured illustrate morphology representative of respective treatments at given times (*n* = 4 dams/treatment/time point). Arrows indicate presence of alveoli. Bars = 100 μm.

In the lactational challenge on PND10, neither milk volume nor timed nursing behavior was significantly different from controls with gestational (P_0_) or chronic, low-level PFOA exposure of the F_1_ dams (Table 3). Although we noted large differences in mean values (i.e., one-third reduction in milk transferred to offspring as measured by litter weight and an 84-sec longer time to suckling in the 1-mg/kg + 5 ppb PFOA exposure group compared with controls), high variability in these responses limited the power to detect a significant difference. Nevertheless, F_1_ lactational morphology was significantly compromised among all treatment groups at PND10 (Table 3, Figure 2). By PND22, most morphological delays were no longer evident, and only F_1_ dams with the highest developmental exposure (i.e., 5 mg/kg PFOA) still exhibited morphology that was significantly different from controls, with little evidence of normal regression. Consistent with this, we observed productive spherical alveoli in the 5 mg/kg group, in contrast with the regressing alveoli and apoptotic bodies observed in controls. Of note, at the time F_1_ dams became pregnant and underwent lactational differentiation, their virgin siblings still exhibited stunted mammary gland development in all exposure groups compared with controls (PND63; Table 2, Figure 1).

Despite striking morphological abnormalities in the lactating glands of PFOA-exposed F_1_ dams on PND10, we found no clear evidence of diminished nutritional support provided by these dams based on F_2_ body weights (Table 4). These data suggest that nursing behavior of the neonates may have changed (i.e., increased number of nursing events per day or longer nursing per event) to compensate for the decreased potential in milk production by the F_1_ dam, but we did not evaluate these end points in this study. Adjusted body weights and liver:body weight ratios did not demonstrate clear differences by treatment group in the F_2_ offspring (Table 4).

**Table 4:** F_2_ developmental indices (mean ± SE; *n* = 4–10).

Developmental index	Control	Control + 5 ppb PFOA	1 mg/kg	1 mg/kg + 5 ppb PFOA	5 mg/kg
Body weight (g)										
PND1		1.71 ± 0.03		1.61 ± 0.03*		1.63 ± 0.05		1.68 ± 0.05		1.65 ± 0.04
PND3		2.27 ± 0.05		2.22 ± 0.05		2.25 ± 0.09		2.30 ± 0.09		2.22 ± 0.06
PND5		3.24 ± 0.07		3.35 ± 0.10		3.38 ± 0.11		3.42 ± 0.15		3.34 ± 0.09
PND10		5.69 ± 0.22		5.83 ± 0.23		6.00 ± 0.19		5.96 ± 0.18		5.87 ± 0.20
PND14		6.26 ± 0.06		6.34 ± 0.05		7.30 ± 0.25*		7.54 ± 0.33		6.85 ± 0.26
PND17		6.64 ± 0.13		7.05 ± 0.06		8.15 ± 0.31*		8.19 ± 0.39		7.42 ± 0.37
PND22		10.80 ± 0.28		11.41 ± 0.26		13.00 ± 0.50*		13.29 ± 0.61		11.60 ± 0.54
Liver:body weight ratio (×100%)										
PND10		2.94 ± 0.15		2.94 ± 0.12		3.08 ± 0.14		2.73 ± 0.14		2.91 ± 0.09
PND22		5.43 ± 0.14		5.25 ± 0.25		5.10 ± 0.21		5.18 ± 0.23		5.11 ± 0.15
PND42		5.43 ± 0.13		5.47 ± 0.10		5.78 ± 0.12		5.36 ± 0.19		5.63 ± 0.21
PND63		5.28 ± 0.25		5.13 ± 0.19		5.05 ± 0.11		5.10 ± 0.15		4.79 ± 0.25
Body weight excluding liver weight (g)										
PND10		6.20 ± 0.18		6.15 ± 0.20		6.16 ± 0.14		5.72 ± 0.29		6.44 ± 0.36
PND22		9.75 ± 0.58		10.10 ± 0.18		10.58 ± 0.54		11.29 ± 0.73		10.41 ± 0.78
PND42		22.28 ± 0.79		24.07 ± 0.32		24.12 ± 0.68		25.78 ± 0.55*		24.12 ± 0.51
PND63		27.41 ± 0.76		27.59 ± 1.22		25.98 ± 1.29		28.83 ± 0.90		29.66 ± 2.10
Mammary gland score (1–4 scale)										
PND10		2.8 ± 0.3		3.0 ± 0.2		1.9 ± 0.3		2.6 ± 0.2		2.0 ± 0.2
PND22		3.1 ± 0.4		1.9 ± 0.3		2.3 ± 0.1		2.3 ± 0.2		2.0 ± 0.2
PND42		3.5 ± 0.2		2.5 ± 0.4*		3.4 ± 0.2		2.4 ± 0.2*^#^		3.3 ± 0.4
PND63		3.4 ± 0.2		3.5 ± 0.2		2.4 ± 0.2*		2.6 ± 0.5		2.6 ± 0.4
*p < 0.05 compared with control. ^#^*p* < 0.05 compared with 1 mg/kg.										

**Figure 3:**
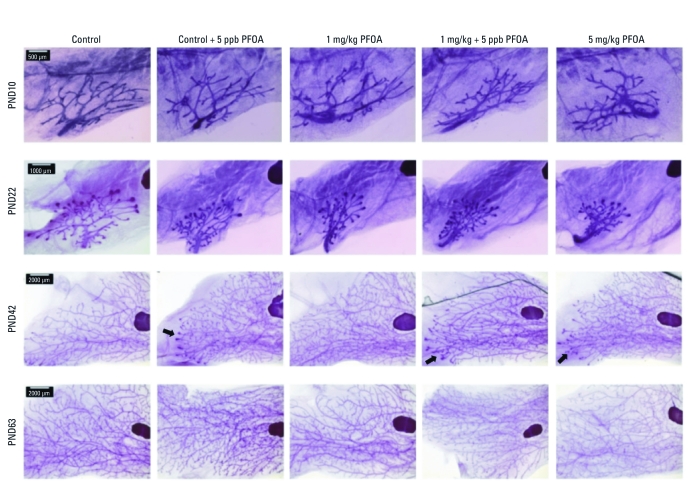
F_2_ female mammary gland development. Mammary whole-mounts illustrate morphology representative of respective treatment groups at PNDs 10, 22, 42, and 63 (*n* = 4–5 females/treatment/age). Arrows indicate remaining TEBs. Bars = 500 μm for PND10, 1,000 μm for PND22, and 2,000 μm for PND42 and PND63.

Unlike F_1_ females, developmental mammary gland scores in F_2_ females did not differ in association with maternal exposure; however, control F_2_ females exhibited unusually low mammary gland scores at PND10 and PND22, which might have reduced the statistical ability to detect effects in other treatment groups at these time points (Table 4). At PND22, scores were consistent with developmental delays in all treatment groups relative to controls, but contrasts were not statistically significant. By PND42, both groups with chronic drinking-water exposures (control + 5 ppb PFOA, 1 mg/kg + 5 ppb PFOA) displayed significantly reduced gland development relative to controls (Table 4) that was characterized by an excess of terminal end buds (TEBs) (Figure 3). Furthermore, mammary gland scores for the F_2_ offspring of gestationally exposed F_1_ females in the 5 mg/kg group were generally consistent with delayed differentiation (Table 4), with histological evidence of postponed lobule formation (arrows in Figure 3). We frequently observed a more sparse appearance in F_2_ mammary tissue from these three groups (data not shown), resulting from delayed ductal outgrowth and persistence of TEBs in adults (arrows in Figure 3).

*Water consumption.* We calculated average daily PFOA intake for the two groups receiving chronic drinking-water exposures from measurements of weekly water consumption [see Supplemental Material, Figure 2 (doi:10.1289/ehp.1002741)]. We found no difference in water intake between groups (as a function of P_0_ treatment), and daily estimated PFOA intake for drinking-water groups ranged from approximately 50 to 100 ng, excepting anticipated changes in water intake depending on life stage (i.e., increased intake during lactation, lower intake in early life; see Supplemental Material, Table 1 and Supplemental Material, Figure 2).

*Serum PFOA analyses.* In F_1_ offspring at 9 weeks of age (PND63; Table 5), serum PFOA concentrations in the 5 mg/kg group were only an order of magnitude greater than the levels exhibited in the chronic drinking water + 5 ppb PFOA exposure-only group. When F_1_ dams (then 13 weeks of age) were weaning their litters (F_2_ at PND22; Table 5), serum PFOA concentrations in the F_2_ drinking-water exposure groups had surpassed those of the F_2_ offspring of F_1_ dams developmentally exposed to 1 and 5 mg/kg  PFOA during their gestation. The control + 5 ppb PFOA group was particularly interesting, because averaged over their lifetimes (PNDs 22, 42, and 63, and ~ PND91 for F_1_ dams, means averaged for each respective generation), the F_1_ and F_2_ generations exhibited nearly identical average serum PFOA concentrations, 59.5 and 50.8 ng/mL, respectively. Furthermore, because the final serum measurement taken on the F_1_ generation was at 13 weeks postnatally (~ PND91), compared with only 9 weeks for the F_2_ generation, the lifetime average may have been skewed slightly higher for the F_1_ generation. Serum PFOA concentrations did not differ significantly at any time point between the two drinking-water treatment groups in the F_2_ generation.

**Table 5:** Serum PFOA concentrations (ng/mL) over three generations (mean ± SE).

Generation/age	Control	Control + 5 ppb PFOA	1 mg/kg	1 mg/kg + 5 ppb PFOA	5 mg/kg
P_0_ dams at weaning (PND22)		4.0 ± 0.3		74.8 ± 11.3		6658.0 ± 650.5		4772.0 ± 282.4		26980.0 ± 1288.2
F_1_ pups										
PND22		0.6 ± 0.3		21.3 ± 2.1		2443.8 ± 256.4		2743.8 ± 129.4		10045 ± 1125.6
PND42		1.4 ± 0.4		48.9 ± 4.7		609.5 ± 72.2		558.0 ± 55.8		1581.0 ± 245.1
PND63		3.1 ± 0.2		66.2 ± 4.1		210.7 ± 21.9		187.0 ± 24.1		760.3 ± 188.3
F_1_ dams at weaning (PND22)		2.0 ± 0.6		86.9 ± 14.5		9.3 ± 2.6		173.3 ± 36.4		18.7 ± 5.2
F_2_ pups										
PND22		0.4 ± 0.0		26.6 ± 2.4		4.6 ± 1.2		28.5 ± 3.7		7.8 ± 1.9
PND42		0.7 ± 0.3		57.4 ± 2.9		0.4 ± 0.0		72.8 ± 5.8		0.4 ± 0.0
PND63		1.1 ± 0.4		68.5 ± 9.4		1.1 ± 0.5		69.2 ± 4.3		1.2 ± 0.5

## Discussion

Our prior studies identified morphological delays in mammary gland development that resulted from gestational PFOA exposure (White et al. 2007, 2009), but we did not previously determine whether such morphological effects persisted and were associated with functional consequences, nor did we evaluate the effects of low-level, chronic exposures, similar to nonoccupational exposures in humans. In the present study, we found evidence that the previously reported effects on F_1_ offspring mammary development—resulting from treatment of P_0_ dams with 1 or 5 mg/kg PFOA during pregnancy—did persist and that these histopathological diminishments in the developing gland translated to altered lactational morphology, when F_1_ females were bred and challenged to lactationally support F_2_ litters. However, these effects were not associated with an overt reduction in the nutritional support provided by the F_1_ dam, because F_2_ offspring demonstrated normal postnatal survival and weight gain. Among F_1_ females that received only chronic low-level 5 ppb PFOA exposure, we also observed comparable and significant diminishments in developmental morphology between PND22 and PND63, as well as in later, adult lactational morphology at the peak of lactation, suggesting a far greater sensitivity of the tissue than previously identified. F_2_ offspring of these F_1_ dams with only chronic low-dose exposures also displayed a trend toward delayed development and exhibited significantly stunted morphology at PND42.

The degree to which these persistent alterations in F_1_ mammary gland morphology are associated with functional consequences is difficult to determine because impaired weight gain in F_2_ offspring was the only relevant end point assessed. The morphological effects of PFOA exposure in F_1_ mammary glands did not translate to significant decreases in growth and survival of F_2_ litters, as opposed to the case with F_1_ offspring of P_0_ dams. Nonetheless, an increase in the thriftiness of offspring from the F_1_ to F_2_ generations or an increase in F_2_ nursing frequency could have masked effects on milk production in affected lactating F_1_ glands.

These data suggest that chronic developmental exposure to environmentally relevant levels of PFOA may not interfere with lactation per se, but may reduce the number and density of alveoli available to produce milk and increase latency to peak milk output, delaying offspring maturation as seen in our previous work (White et al. 2007). In the case of humans, where viable alternatives to breast milk are available, low-level functional effects on lactation that cause even a short delay in substantial milk output might result in formula feeding instead of breast-feeding, despite the established health benefits of breast-feeding. In mammalian wildlife species, critically reliant upon lactation to raise their offspring, responsiveness of the gland to PFOA might lead to delays in milk production, resulting in malnourishment or possibly starvation of offspring, in a manner similar to the effects of polychlorinated biphenyls on wild mink reproduction in the past (Aulerich and Ringer 1977).

Chronic, low-dose PFOA exposure in drinking water at human-relevant levels (5 ppb) delayed mammary gland development in F_1_ offspring. This exposure yielded serum PFOA levels that ranged between 50 and 100 ng/mL after approximately 6 weeks (Table 5; lifetime averages: F_1_ females, 59.5 ng/mL; F_2_ females, 50.8 ng/mL; data not shown). If these approximate serum concentration ranges represent those of an animal reaching a steady-state burden, it should be noted that they are approximately an order of magnitude lower than that seen in some chronically exposed human populations. For example, communities exposed to 3.55 ppb PFOA in municipal supply drinking water exhibited mean serum PFOA concentrations of 423 ng/mL (Emmett et al. 2006), compared with the national average of 3.9 ng/mL (Calafat et al. 2007). Although it is understood that the pharmacokinetics of PFOA in the mouse differ from those in the human—the half-life being approximately 17 days in the mouse and 3.8 years in the human (Calafat et al. 2007; Lou et al. 2009)—it remains disconcerting that the effective circulating dose sufficient to yield histopathological changes in the mouse mammary gland is approximately an order of magnitude lower than the mean serum concentration in certain human populations.

These low serum concentrations were associated with alterations in mouse mammary gland morphology in three generations, although we could not separate the effects of postgestational chronic exposure in each generation from gestational exposure in some instances, so the effects observed in these treatment groups were not necessarily transgenerationally transmitted. Because humans with exposures under similar conditions (contaminated drinking water) exhibit higher circulating serum concentrations of PFOA, by an order of magnitude—and approximately two orders of magnitude above the concentration of PFOA in their exposure source—the data presented here may actually underrepresent human-relevant exposure conditions with respect to internal circulating dose. However, it is not known whether effects of PFOA on the mouse mammary gland translate to effects in humans; research is ongoing to discern a mammary-specific mode of action for PFOA and to determine its relevance to human breast health.

## Conclusion

Our studies identified a gestational exposure lowest observable adverse effect level (LOAEL) of 1 mg/kg PFOA for altered lactational morphology in treated P_0_ dams and altered mammary gland development in their F_1_ offspring. Additionally, our use of a nontraditional treatment regimen using low-dose continual exposure has generated data that will allow others to calculate a lower chronic exposure LOAEL or benchmark dose.

Delays in mammary epithelial growth in F_1_ females developmentally exposed to PFOA reported in this study and others (White et al. 2007, 2009) translated to histopathological changes in subsequent lactational morphology. However, this did not result in functional deficits in lactation when F_2_ offspring growth and survival were used as proxy measures of nutritional support. We observed sparse branching morphology and delayed differentiation in three generations of CD-1 mice, but the global scoring method did not indicate consistent differences from controls across F_2_ time points.

Although the chronic low-dose PFOA supplied in drinking water in these studies and similar concentrations reported in municipal drinking-water supplies near fluorochemical plants are not representative of drinking- water supplies in the United States in general, PFOA is not regularly monitored in drinking water, so national averages cannot be well estimated. It is concerning, however, that the chronic low dose employed here was sufficient to produce changes in the development of the mouse mammary gland; similar developmental changes are physiologically possible in girls but would likely not be realized until they enter puberty or attempt lactation. Therefore, if human exposures in distinct populations are approximating those provided in this study, concerns over human breast health and lactational competency are justified.

## Supplemental Material

(608 KB) PDF
