# Supplementary material for: Gestational and Chronic Low-Dose PFOA Exposures and Mammary Gland Growth and Differentiation in Three Generations of CD-1 Mice
Source: Environ Health Perspect. 2011 Apr 18;119(8):1070–6. doi: 10.1289/ehp.1002741 (PMC3237341; doi:10.1289/ehp.1002741)
Supplement: (608 KB) PDF [file ehp.1002741.s001.pdf]

Supplemental Material for Environmental Health Perspectives

Gestational and Chronic Low-Dose PFOA Exposures and Mammary Gland Growth and Differentiation in Three Generations of CD-1 Mice.

Sally S. White[1], Jason P. Stanko[1], Kayoko Kato[2], Antonia M. Calafat[2], Erin P. Hines[3], Suzanne E. Fenton[1]

[1] National Toxicology Program, National Institute of Environmental Health Sciences, National Institutes of Health, Research Triangle Park, NC, USA

[2] Division of Laboratory Science, National Center for Environmental Health, Centers for Disease Control and Prevention, Atlanta, GA, USA

[3] National Center for Environmental Assessment, Office of Research and Development, U.S. Environmental Protection Agency, Research Triangle Park, NC, USA.

Address correspondence to:
Dr. Suzanne Fenton
P.O. Box 12233
Bldg 101, Mail Drop E1-08
Research Triangle Park, NC 27709
Phone: (919) 541-4141
Email: fentonse@niehs.nih.gov

Supplemental Material:

- Figure 1. Study design and experimental timeline.
- Figure 2. Average daily PFOA consumption on a weekly-basis.
- Table 1. Estimated average doses of PFOA for treated P0 dams.

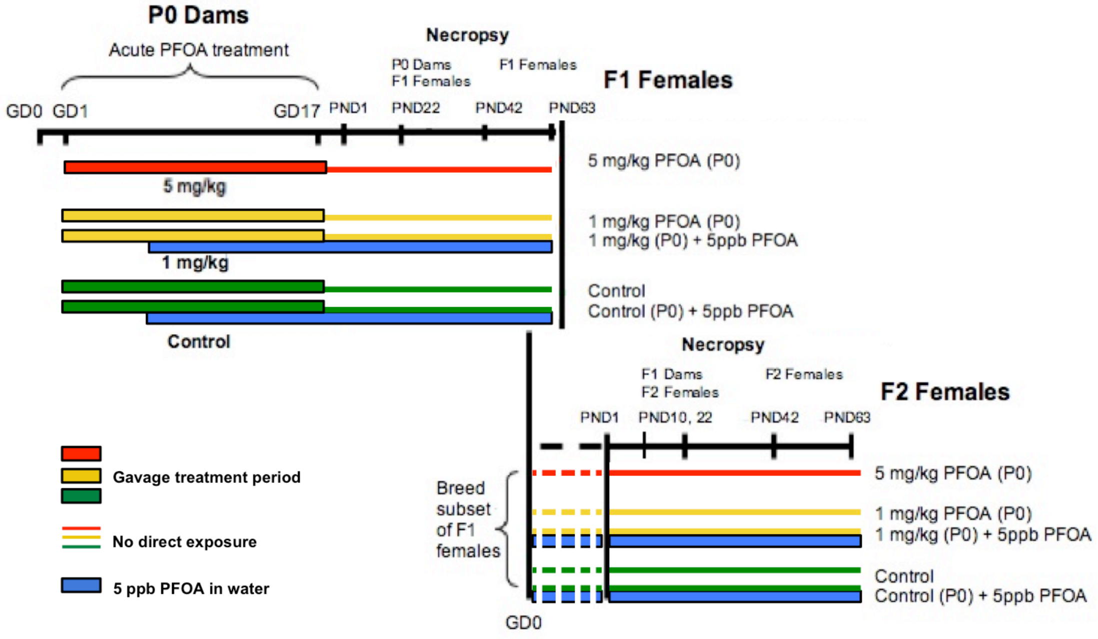

Supplemental Material, Figure 1. Study design and experimental timeline. Bar color denotes dose – green, 0 mg PFOA/kg body weight/day; yellow, 1 mg PFOA/kg body weight/day; red, 5 mg PFOA/kg body weight /day; blue, 5 ppb PFOA in drinking water – and bar thickness denotes timing of treatment – thick bars denote on-going direct treatment, thin bars denote only group identity subsequent to treatment.

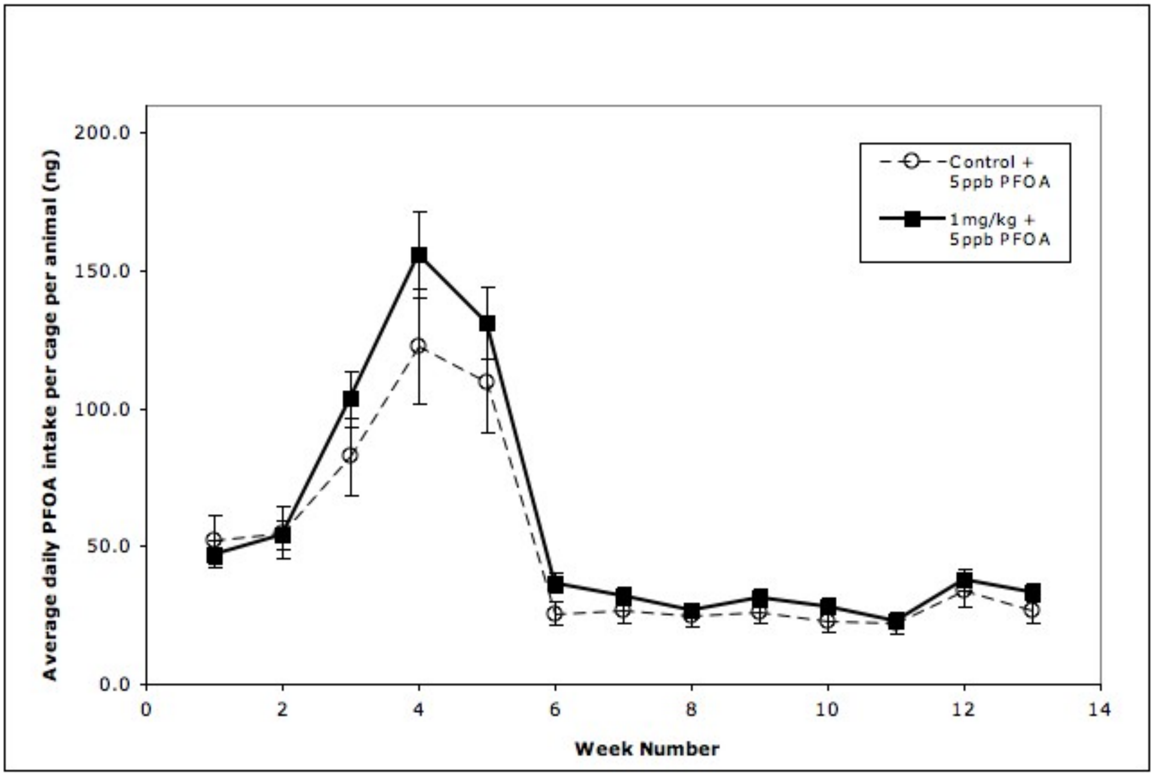

Supplemental Material, Figure 2. Average daily PFOA consumption on a weekly-basis. PFOA consumption was calculated based on water consumption over the course of a week. For each week an average daily intake for the animals in a given cage was calculated, which was then averaged for the treatment group. Here, the first week of drinking water exposure was initiated on GD7 for P0 dams. Thus, parturition (PND1) occurred approximately after week number 2, before consumption spiked. Lactation occurred during weeks 3-5 (until PND22), when water intake spiked to support fluid needs for milk production. After week 4, intake fell some as the litters began to slowly wean and involution started, reducing milk production. At the end of week 5, all P0 dams were euthanized, so only immature F1 offspring remained at week 6 and later, which had lower water intake than did the mature P0 dams. After weaning, however, as F1 offspring matured between weeks 6 and 13, intake increased slowly. Error bars represent +/- SE.

Supplemental Material, Table 1. Estimated average doses of PFOA for treated P0 dams.

	PFOA exposure parameters				
	Control	Control + 5 ppb PFOA	1 mg/kg	1 mg/kg + 5 ppb PFOA	5 mg/kg
Dose indices					
Approximate average daily dose during gestation	0 µg	00.054 µg	36 µg	37 + 0.051 µg	187 µg
Approximate average daily dose during lactation (PND1-22)	0 µg	0.105 µg	0 µg	0 + 0.130 µg	0 µg
Approximate average cumulative dose on PND 1	0 µg	0.648 µg	615 µg	625 + 0.612 µg	3,179 µg
Approximate average cumulative dose on PND 22	0 µg	2.958 µg	615 µg	625 + 3.472 µg	3,179 µg

Estimates of average daily dose from chronic water exposures (Control + 5 ppb PFOA, 1mg/kg + 5ppb) are calculated based on total weekly water consumption, divided by the number of days per week. Cumulative doses from chronic water exposures are calculated based on average daily doses during the given period, multiplied by the number of days in the period. Note, the P0 dams did not receive PFOA in drinking water until GD 7. Thus, the approximate average daily dose during gestation applies for only 12 days (GD7-18), while the approximate average daily dose during lactation applies for 22 days (PND1-22). Average daily dose from acute exposures (1mg/kg PFOA, 5mg/kg PFOA) are calculated based on daily volume administered, as a function of body weight, over the 17-day dosing period. Cumulative doses from acute exposures are calculated by summing the daily administered doses over the treatment period (GD1-17). Note, for this reason cumulative dose does not increase between PND1 and PND22. (N = 7-11 dams).
